# Empirical and process-based approaches to climate-induced forest mortality models

**DOI:** 10.3389/fpls.2013.00438

**Published:** 2013-11-13

**Authors:** Henry D. Adams, A. Park Williams, Chonggang Xu, Sara A. Rauscher, Xiaoyan Jiang, Nate G. McDowell

**Affiliations:** ^1^Earth and Environmental Sciences Division, Los Alamos National LaboratoryLos Alamos, NM, USA; ^2^Theoretical Division, Los Alamos National LaboratoryLos Alamos, NM, USA; ^3^Atmospheric Chemistry Division, National Center for Atmospheric ResearchBoulder, CO, USA

**Keywords:** forest mortality, tree mortality mechanism, vegetation change, dynamic global vegetation model (DGVM), earth system model (ESM), biosphere-atmosphere feedbacks, global change

Globally, forests store ~45% of carbon sequestered terrestrially, contribute more to the terrestrial sink per area than any other land cover type, and assimilate an important portion of anthropogenic emissions (Bonan, 2008). Forests exert strong biophysical control on climate via surface energy balance (Bonan, 2008; Rotenberg and Yakir, 2010; Houspanossian et al., 2013), and the hydrological cycle (Zhang et al., 2001; Brown et al., 2005). Widespread forest mortality in response to drought, increased temperatures, and infestation of tree pests has been observed globally, potentially threatening forests' regulation of climate (Kurz et al., 2008; Adams et al., 2010; Allen et al., 2010; Anderegg et al., 2013a). This threat has prompted great interest in understanding and predicting tree mortality due to climate variability and change, especially drought. Initial tests of hydraulic failure (mortality caused by irreversible loss of xylem conductivity from air embolism), carbon starvation (mortality due to carbohydrate limitation), insect attacks, wildfire, and their interdependence (Allen, 2007; McDowell et al., 2008, 2011, 2013a), suggest proximal causes of mortality are likely complex, co-occurring, interrelated, and variable with tree species (supported by Adams et al., 2009, 2013; Sala et al., 2010; Piper, 2011; Zeppel et al., 2011; Anderegg et al., 2012a, 2013b; Adams et al., 2013; Anderegg and Anderegg, 2013; Galvez et al., 2013; Gaylord et al., 2013; Hartmann et al., 2013a,b; Mitchell et al., 2013; Quirk et al., 2013; Williams et al., in review). While the interdependent roles of carbon and water in plant mortality are consistently observed, this work is continuously prompting new questions (Sala et al., 2010; McDowell et al., 2013b; O'Grady et al., 2013).

The justification for physiological research on drought-induced tree mortality is often stated as a need to improve the predictive capability of vegetation models through incorporation of mortality mechanisms (Fisher et al., 2010; McDowell et al., 2011, 2013a; Powell et al., 2013). Yet if mortality is particularly complicated and associated with failure of multiple physiological processes (Manion, 1981; McDowell et al., 2011; Anderegg et al., 2012b), then a key question emerges: is a mechanistic approach necessary for accurate prediction of future mortality? The answer to this question ultimately depends on the application and goal of the model.

At issue is whether increasing model complexity will improve prediction, which is influenced in part by the modeling approach employed. Two endpoints on a theoretical continuum of approach to mechanism are process-based and empirical model types. The process-based approach focuses on simulating detailed physical or biological processes that explicitly describe system behavior, while the empirical approach relies on correlative relationships in line with mechanistic understanding, but without fully describing system behaviors and interactions (Korzukhin et al., 1996; Table 1). Process-based models can be more comprehensive and incorporate mechanism explicitly, while the empirical approach is typically simpler, with mechanism implicit. These approaches are not exclusive model classifications; All process-based models include some empirical information (e.g., in the choice of relevant mechanisms), and the correlative relationships of empirical models assume a link to process (Korzukhin et al., 1996; Makela et al., 2000). Realistically, many models use a hybrid approach, combining process-based and empirical representation of relationships.

**Table 1:** **Relative differences in the characteristics of process-based and empirical modeling approaches**.

	**Process-based**	**Empirical**
Relationship type	Causal	Correlative
Relative comprehensiveness	More comprehensive	Less comprehensive
Incorporation of mechanism	Explicit	Implicit
Primary source of error	Unknown parameters and processes	Extrapolation
Model uncertainty	Higher	Lower
Data requirements	Higher	Lower
Spatial scale for calibration	Smaller	Smaller to larger
Spatial scaling of prediction	Smaller to Larger	Best at scale of calibration

The advantages and disadvantages of both approaches have been well acknowledged in ecology (Korzukhin et al., 1996; Levin et al., 1997; Makela et al., 2000; Green et al., 2005; Van Nes and Scheffer, 2005). Uncertainty in process-based model outputs could be higher than for the empirical approach due to greater model parameters and data inputs to represent the many processes in the system (Table 1). In the empirical approach, model uncertainty may be reduced, yet significant bias can result from exclusion of important system components by extrapolation of correlative relationships beyond observed variability. Process-based models can better include novel or no-analog responses, those which may occur with future conditions but are not well quantified in past observations (Williams and Jackson, 2007). Ensembles of multiple models are often implemented in climate prediction (Jones, 2013), and can be used to reduce uncertainty in biological responses to climate change (Asseng et al., 2013). An ensemble approach for forest mortality should include models from across the spectrum of empirical to process-based types (e.g., McDowell et al., 2013a). In previous decades, process-based modeling was often limited by computing power, but improvement is now frequently limited by availability of data needed to initialize, parameterize, and evaluate models (Hall, 1988; Onstad, 1988; Levin et al., 1997; McDowell et al., 2011).

The decision between using relatively process-based or empirical approaches also depends on the scale of spatial and temporal inference. For example, to quantify the feedback of vegetation impacts upon future climate, reliable predictions of forest mortality at the global scale should be simulated. However, most field studies have focused on small scales. Therefore, techniques to extend small-scale understanding to large-scale models are critically needed (McDowell et al., 2013a). Models developed at the plot scale often have data input requirements that are not available at larger scales. This is a particularly important problem for more process-based models, which typically require more data inputs (Table 1). In contrast, because the mechanisms leading to mortality could differ among regions and species, relatively empirical models developed for one location/region or species may not be applicable to another, necessitating development for each region or species. Process-based models are more robust for scaling across regions and species due to the coupled representation of multiple basic processes; however, model simplification may be necessary in order to make the large-scale simulation feasible in respect to computational cost and data requirements.

Both process- and empirically-based models suffer from two large data-gaps. First, most mortality studies have focused on a few species, but for a global simulation, comprehensive representation of many species across different regions is required. This has been achieved by grouping species into functional types (Woodward and Cramer, 1996), but future research should refine these groups based on empirically determined links between species drought strategies and mechanisms of mortality. Second, to evaluate models at different scales, it is important that we have comprehensive mortality benchmarking datasets across different regions and functional types. Currently, few such datasets are available, substantially limiting mortality model progress at large scales (Allen et al., 2010).

The challenge of bridging mechanism and scales is arguably greatest at the global scale, where finer scale processes (e.g., photosynthesis) must be simulated across the Earth. Dynamic global vegetation models (DGVMs) coupled with general circulation models are a common tool for simulating vegetation response to climate change (Sitch et al., 2008; Jiang et al., 2013). Early DGVMs that specifically included forest mortality represented mortality simply, with routines representing the stem exclusion phase (intra-species competition) of forest stand development and/or dependent on minimum productivity thresholds for tree survival (Bugmann, 2001; Cox, 2001; Sitch et al., 2003, 2008; see also Box 1 in McDowell et al., 2011). There has been increased effort toward representing forest mortality in DGVMs with more detail using process-based approaches. In a recent version of the Community Land Model with dynamic vegetation enabled (CLM4-CNDV), which is based on the Lund-Potsdam-Jena model (Sitch et al., 2003), vegetation represented by plant functional types is established and changes according to biogeography rules based on temperature thresholds and a minimum precipitation requirement (Levis et al., 2004; Oleson et al., 2010). Annual mortality can occur in the model due to light, competition, fire, growth efficiency, and heat-stress tolerance (Levis et al., 2004; Jiang et al., 2013; Figure 1). The Ecosystem Demography model (Moorcroft et al., 2001) has been updated with algorithms for tree carbon resources and xylem cavitation to represent carbon starvation and hydraulic failure mechanisms (Fisher et al., 2010; McDowell et al., 2013a). Other process-based models not linked to DGVMs have been developed to predict tree mortality at stand to regional scales. These include TREES, which simulates mortality from gas exchange, soil-plant hydraulics, and carbohydrate dynamics (Loranty et al., 2010; Mackay et al., 2012; McDowell et al., 2013a), and LANDIS-II, a forest succession model extended to predict mortality from drought duration and intensity (Gustafson and Sturtevant, 2013). Epidemiological models that incorporate tree stress and insect population dynamics have also used a relatively process-based approach (Powell and Bentz, 2009).

**Figure 1:**
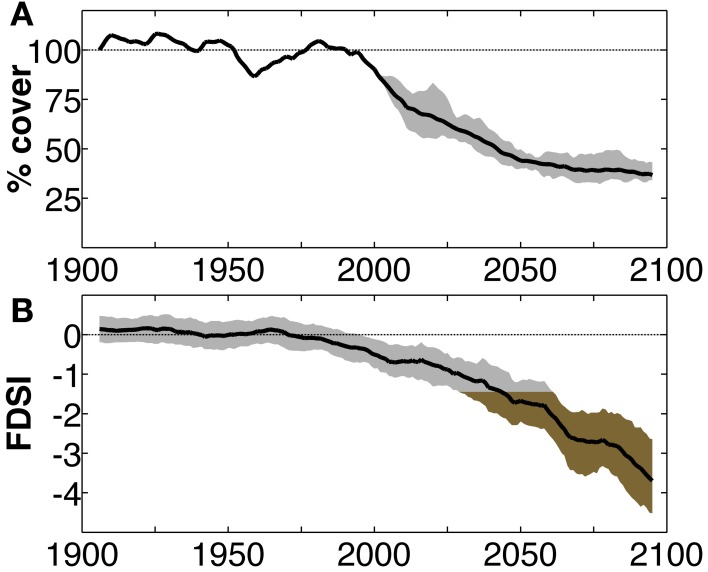
**A comparison of tree mortality projections for the southwest USA from a more process-based Dynamic Global Vegetation Model, and a less process-based, empirical index that could be applied in a mortality model. (A)** Projected heat-induced decline in relative conifer forest cover using the Community Land Model with dynamic vegetation enabled (CLM4-CNDV; Jiang et al., 2013), with the ability to account for the impact of declining forest population on mortality rate. Shading bounds eight projections assuming unique model-projected scenarios of global sea surface temperatures. Black line: ensemble mean. **(B)** Forest Drought Severity Index (FDSI) predicted through 2100 by applying an empirical growth-climate relationship to modeled projections of precipitation and vapor-pressure deficit (Williams et al., 2013). More negative values indicate intensified forest stress. Shading bounds the inner quartiles of CMIP3 model projections. Brown shading represents FDSI values more severe than the estimated regional mortality threshold (−1.41, average FDSI of the most severe 50% of years during the 1573–1587 megadrought). Black line: ensemble mean. Note the inflection point in the decline of forest cover in **(A)** corresponds with FDSI crossing the regional mortality threshold (2030–2060) in **(B)**. Both imply similar predictions for mortality, and each model incorporates information that the other could benefit from.

In contrast to process-based approaches for tree mortality simulation, simpler, more empirical methods could offer valid and rapid alternatives for projection of climate change effects on forests. Manion's 1981 pre-disposing factor framework has been used to predict tree vigor decline that leads to death at the level of individual trees (Güneralp and Gertner, 2007) and vulnerability to insect attack at the stand level (Coops et al., 2009). Xylem resin anatomy, which integrates tree stress and defense against bark beetles, was an effective predictor of individual tree mortality (Kane and Kolb, 2010), and may be a productive avenue for model development using simple climate-xylem resin anatomy relationships from tree-rings. Bioclimatic envelope models are an example of a primarily empirical approach often used to predict tree species response to future climate (e.g., Iverson et al., 1998, 2008; Rehfeldt et al., 2012). In these models the relationship between the current climate at species' range limits are used to predict future distributions by moving these species' range limits to match the location of these climate envelopes under future conditions (see Table 1 in Araújo and Peterson, 2012). When appropriately used, this approach projects potential tree species habitat, not actual future distributions (Iverson et al., 2008; Araújo and Peterson, 2012), although similar relationships are used for survival thresholds in Lund-Potsdam-Jena and other DGVMs (Sitch et al., 2003; Jiang et al., 2013). Development of mortality-specific envelopes or empirical indices with climatic and environmental (e.g., soil moisture) thresholds for tree survival that have been directly tested or observed would improve predictions based on these relationships.

There can be different levels of complexity in such empirical approaches. For example, the forest drought stress index (FDSI), derived from tree-ring records and predictable from regional climate, is highly correlated with forest mortality in the southwest USA (Williams et al., 2013, Figure 1). Williams et al. infer FDSI through AD 2100 from climate projections and empirically predict that by mid-century the southwest USA will be less suitable for forests than in at least 1000 years due to increasing atmospheric moisture demand. The timing of mortality implied by the FDSI projection coincides with the period of rapid forest loss predicted in recent simulations using the Community Land Model coupled with nitrogen and vegetation dynamics (CLM4-CNDV; Jiang et al., 2013). The CLM4-CNDV uses an empirical approach of mortality, including thresholds of growth rate and heat stress, and moisture limitations, but relies on explicit process simulation including photosynthetic simulation for vegetation growth (Jiang et al., 2013; Figure 1). The relationship between FDSI and recent tree mortality rates (see Figure 2 in Williams et al., 2013) suggests a potentially simpler empirical application for this stress index in a mortality model. The FDSI approach requires development of empirical climate-tree stress and mortality relationships for a specific region, while the dynamic vegetation approach of Jiang et al. (2013) simulates universal forest interactions with climate. Although the DGVM is much more complex than the empirical relationships in an FDSI approach, results from both are similar for the southwest USA (Figure 1). Thus, the empirical approaches like FDSI are simple but powerful, and regionally based empirical results may inform DGVMs regarding climatic threshold effects on vegetation.

With an imperfect understanding of the physiological processes involved, we currently cannot discern the causes of tree mortality from the symptoms of dying. For example, it is not known whether the carbohydrate depletion observed as some trees die from drought may be a resultant symptom of mortality by hydraulic failure, or a directly contributing cause of mortality (McDowell et al., 2011; Adams et al., 2013). Due to these limitations, earth system models should take greater advantage of empirical relationships between climate and forest mortality to bridge knowledge gaps in mechanistic understanding, as global climate projections that incorporate biophysical feedbacks from forest loss are urgently needed for policy decisions. We encourage use of hybrid models and model ensembles that span the empirical to process-based continuum of approaches. Relatively empirical approaches, such as models based on FDSI-mortality correlations, could provide for rapid model development in tree mortality prediction.

Nevertheless, we do not advocate ceasing process-based model development. Inclusion of process-based representation for tree mortality mechanism has the potential to deliver more accurate projections if causal relationships are better understood. Moreover, even if parameterization for global process-based models is not possible in the near future, process-based models at fine scales may inform development of broader-scale empirical models through their ability to account for drought-driven changes in forest composition and distribution. We suggest several steps towards development of process-based, mechanistic models: (1) improved experimentation to distinguish physiological causes from symptoms, (2) continued model development based on existing knowledge and emerging discovery, (3) improved model validation against both experimental results and regional-scale mortality observations, and (4) high-resolution measurement of forest composition at large scales. While ecologists studying tree mortality have favored investigating physiological mechanism, measuring species composition and mortality at high resolution across regions is crucial for providing baseline observations to constrain model predictions. Without improved input on current forest conditions, even accurate models of forest mortality cannot generate useful predictions of change.

## References

[B1] Adams H. D. Guardiola-Claramonte M. Barron-Gafford G. A. Villegas J. C. Breshears D. D. Zou C. B. (2009). Temperature sensitivity of drought-induced tree mortality portends increased regional die-off under global-change-type drought. Proc. Natl. Acad. Sci. U.S.A. 106, 7063–7066 10.1073/pnas.0901438106 19365070 PMC2678423

[B2] Adams H. D. Macalady A. K. Breshears D. D. Allen C. D. Stephenson N. L. Saleska S. R. (2010). Climate-induced tree mortality: earth system consequences. Eos Trans. Am.Geophys. Union 91, 153–154 10.1029/2010EO170003

[B3] Adams H. D. Germino M. J. Breshears D. D. Barron-Gafford G. A. Guardiola-Claramonte M. Zou C. B. (2013). Nonstructural leaf carbohydrate dynamics of Pinus edulis during drought-induced tree mortality reveal role for carbon metabolism in mortality mechanism. New Phytol. 197, 1142–1151 10.1111/nph.12102 23311898

[B4] Allen C. D. (2007). Interactions across spatial scales among forest dieback, fire, and erosion in northern New Mexico landscapes. Ecosystems 10, 797–808 10.1007/s10021-007-9057-4

[B5] Allen C. D. Macalady A. K. Chenchouni H. Bachelet D. McDowell N. Vennetier M. (2010). A global overview of drought and heat-induced tree mortality reveals emerging climate change risks for forests. For. Ecol. Manage. 259, 660–684 10.1016/j.foreco.2009.09.001

[B6] Anderegg W. R. L. Berry J. A. Smith D. D. Sperry J. S. Anderegg L. D. L. Field C. B. (2012a). The roles of hydraulic and carbon stress in a widespread climate-induced forest die-off. Proc. Natl. Acad. Sci. U.S.A. 109, 233–237 10.1073/pnas.1107891109 22167807 PMC3252909

[B7] Anderegg W. R. L. Berry J. A. Field C. B. (2012b). Linking definitions, mechanisms, and modeling of drought-induced tree death. Trends Plant Sci. 17, 693–700 10.1016/j.tplants.2012.09.006 23099222

[B8] Anderegg W. R. L. Anderegg L. D. L. (2013). Hydraulic and carbohydrate changes in experimental drought-induced mortality of saplings in two conifer species. Tree Physiol. 33, 252–260 10.1093/treephys/tpt016 23514762

[B9] Anderegg W. R. L. Kane J. M. Anderegg L. D. L. (2013a). Consequences of widespread tree Mortality triggered by drought and temperature stress. Nat. Clim. Change 3, 30–36 10.1038/nclimate1635

[B10] Anderegg W. R. L. Plavcova L. Anderegg L. D. L. Hacke U. G. Berry J. A. Field C. B. (2013b). Drought's legacy: multiyear hydraulic deterioration underlies widespread aspen forest die-off and portends increased future risk. Glob. Change Biol. 19, 1188–1196 10.1111/gcb.12100 23504895

[B11] Asseng S. Ewert F. Rosenzweig C. Jones J. W. Hatfield J. L. Ruane A. C. (2013). Uncertainty in simulating wheat yields under climate change. Nat. Clim. Change 3, 827–832 10.1038/nclimate191621049871

[B12] Araújo M. B. Peterson A. T. (2012). Uses and misuses of bioclimatic envelope modeling. Ecology 93, 1527–1539 10.1890/11-1930.1 22919900

[B13] Bonan G. B. (2008). Forests and climate change: forcings, feedbacks, and the climate benefits of forests. Science 320, 1444–1449 10.1126/science.1155121 18556546

[B14] Brown A. E. Zhang L. McMahon T. A. Western A. W. Vertessy R. A. (2005). A review of paired catchment studies for determining changes in water yield resulting from alterations in vegetation. J. Hydrol. 310, 28–61 10.1016/j.jhydrol.2004.12.010

[B15] Bugmann H. (2001). A review of forest gap models. Clim. Change 51, 259–305 10.1023/A:1012525626267

[B16] Coops N. C. Waring R. H. Wulder M. A. White J. C. (2009). Prediction and assessment of bark beetle-induced mortality of lodgepole pine using estimates of stand vigor derived from remotely sensed data. Remote Sens. Environ. 113, 1058–1066 10.1016/j.rse.2009.01.013

[B17] Cox P. M. (2001). Description of the “TRIFFID” Dynamic Global Vegetation Model. Hadley Centre Technical Note No. 24 (Bracknell: UK Met Office).

[B18] Fisher R. McDowell N. Purves D. Moorcroft P. Sitch S. Cox P. (2010). Assessing uncertainties in a second-generation dynamic vegetation model caused by ecological scale limitations. New Phytol. 187, 666–681 10.1111/j.1469-8137.2010.03340.x 20618912

[B19] Galvez D. A. Landhausser S. M. Tyree M. T. (2013). Low root reserve accumulation during drought may lead to winter mortality in poplar seedlings. New Phytol. 198, 139–148 10.1111/nph.12129 23347066

[B20] Gaylord M. L. Kolb T. E. Pockman W. T. Plaut J. A. Yepez E. A. Macalady A. K. (2013). Drought predisposes piñon-juniper woodlands to insect attacks and mortality. New Phytol. 567–578 10.1111/nph.12174 23421561

[B21] Green J. L. Hastings A. Arzberger P. Ayala F. J. Cottingham K. L. Cuddington K. (2005). Complexity in ecology and conservation: mathematical, statistical, and computational challenges. Bioscience 55, 501–510 10.1641/0006-3568(2005)055[0501:CIEACM]2.0.CO;2

[B22] Güneralp B. Gertner G. (2007). Feedback loop dominance analysis of two tree mortality models: relationship between behavior. Tree Physiol. 27, 269–280 10.1093/treephys/27.2.269 17241969

[B23] Gustafson E. J. Sturtevant B. R. (2013). Modeling forest mortality caused by drought stress: implications for climate change. Ecosystems 16, 60–74 10.1007/s10021-012-9596-1

[B24] Hall C. A. S. (1988). An assessment of several of the historically most influential theoretical models used in ecology and of the data provided in their support. Ecol. Modell. 43 10.1016/0304-3800(88)90070-1

[B25] Hartmann H. Ziegler W. Kolle O. Trumbore S. (2013a). Thirst beats hunger—declining hydration during drought prevents carbon starvation in Norway spruce saplings. New Phytol. 200, 340–349 10.1111/nph.12331 23692181

[B26] Hartmann H. Ziegler W. Trumbore S. (2013b). Lethal drought leads to reduction in nonstructural carbohydrates in Norway spruce tree roots but not in the canopy. Funct. Ecol. 12, 1779–1791 10.1111/1365-2435.12046

[B27] Houspanossian J. Nosetto M. Jobbagy E. G. (2013). Radiation budget changes with dry forest clearing in temperate Argentina. Glob. Change Biol. 19, 1211–1222 10.1111/gcb.12121 23504897

[B28] Iverson L. R. Prasad A. M. Matthews S. N. Peters M. (2008). Estimating potential habitat for 134 eastern US tree species under six climate scenarios. For. Ecol. Manage. 254, 390–406 10.1016/j.foreco.2007.07.023

[B29] Iverson L. R. Prasad A. M. Pm (1998). Predicting abundance of 80 tree species following climate change in the eastern United States. Ecol. Monogr. 68, 465–485 10.1890/0012-9615(1998)068[0465:PAOTSF]2.0.CO;2

[B30] Jiang X. Rauscher S. Ringler T. Lawrence D. Williams A. Allen C. (2013). Projected future changes in vegetation in western North America in the twenty-first century. J. Clim. 26, 3672–3687 10.1175/JCLI-D-12-00430.1

[B31] Jones N. (2013). Climate assessments: 25 years of the IPCC. Nature 501, 298–299 10.1038/501298a 24048050

[B32] Kane J. M. Kolb T. E. (2010). Importance of resin ducts in reducing ponderosa pine mortality from bark beetle attack. Oecologia 164, 601–609 10.1007/s00442-010-1683-4 20556621

[B33] Korzukhin M. D. TerMikaelian M. T. Wagner R. G. (1996). Process versus empirical models: which approach for forest ecosystem management. Can. J. For. Res. 26, 879–887 10.1139/x26-096

[B34] Kurz W. A. Dymond C. C. Stinson G. Rampley G. J. Neilson E. T. Carroll A. L. (2008). Mountain pine beetle and forest carbon feedback to climate change. Nature 452, 987–990 10.1038/nature06777 18432244

[B35] Levin S. A. Grenfell B. Hastings A. Perelson A. S. (1997). Mathematical and computational challenges in population biology and ecosystems science. Science 275, 334–343 10.1126/science.275.5298.334 8994023

[B36] Levis S. Bonan G. B. Vertenstein M. Oleson K. (2004). The community land model's dynamic global vegetation model (CLM-DGVM): Technical Description and User's Guide. Techinal Note TN-4591IA. National Center for Atmospheric Research.

[B37] Loranty M. M. Mackay D. S. Ewers B. E. Traver E. Kruger E. L. (2010). Competition for light between individual trees lowers reference canopy stomatal conductance: results from a model. J. Geophys. Res. Biogeosci. 115, G04019 10.1029/2010JG001377

[B38] Makela A. Landsberg J. Ek A. R. Burk T. E. Ter-Mikaelian M. Agren G. I. (2000). Process-based models for forest ecosystem management: current state of the art and challenges for practical implementation. Tree Physiol. 20, 289–298 10.1093/treephys/20.5-6.289 12651445

[B39] Mackay D. S. Ewers B. E. Loranty M. M. Kruger E. L. Samanta S. (2012). Bayesian analysis of canopy transpiration models: a test of posterior parameter means against measurements. J. Hydrol. 432, 75–83 10.1016/j.jhydrol.2012.02.019

[B40] Manion P. D. (1981). Tree Disease Concepts. Englewood Cliffs, NJ: Prentice-Hall

[B41] McDowell N. Pockman W. T. Allen C. D. Breshears D. D. Cobb N. Kolb T. (2008). Mechanisms of plant survival and mortality during drought: why do some plants survive while others succumb to drought. New Phytol. 178, 719–739 10.1111/j.1469-8137.2008.02436.x 18422905

[B42] McDowell N. G. Beerling D. J. Breshears D. D. Fisher R. A. Raffa K. F. Stitt M. (2011). The interdependence of mechanisms underlying climate-driven vegetation mortality. Trends Ecol. Evol. 26, 523–532 10.1016/j.tree.2011.06.003 21802765

[B43] McDowell N. G. Fisher R. Xu C. Domec J. C. Höltta T. Mackay D. S. (2013a). Evaluating theories of drought-induced vegetation mortality using a multimodel-experiment framework. New Phytol. 200, 304–321 10.1111/nph.12465 24004027

[B44] McDowell N. G. Ryan M. G. Zeppel M. J. B. Tissue D. T. (2013b). Improving our knowledge of drought-induced forest mortality through experiments, observations, and modeling. New Phytol. 200, 289–293 10.1111/nph.12502 24050629

[B45] Mitchell P. J. O'Grady A. P. Tissue D. T. White D. A. Ottenschlaeger M. L. Pinkard E. A. (2013). Drought response strategies define the relative contributions of hydraulic dysfunction and carbohydrate depletion during tree mortality. New Phytol. 197, 862–872 10.1111/nph.12064 23228042

[B46] Moorcroft P. R. Hurtt G. C. Pacala S. W. (2001). A method for scaling vegetation dynamics: the ecosystem demography model (ED). Ecol. Monogr. 71, 557–585 10.1890/0012-9615(2001)071[0557:AMFSVD]2.0.CO;2

[B47] O'Grady A. P. Mitchell P. J. M. Pinkard E. A. Tissue D. T. (2013). Thirsty roots and hungry leaves: unraveling the roles of carbon and water dynamics in tree mortality. New Phytol. 200, 294–297 10.1111/nph.12451 24050630

[B48] Oleson K. W. Lawrence D. M. Bonan G. B. Flanner M. G. Kluzek E. Lawrence P. J. (2010). Technical Description of version 4.0 of the Community Land Model (CLM). NCAR Technical Note NCAR/TN-478+STR. National Center for Atmospheric Research. 10.5065/D6FB50WZ

[B49] Onstad D. W. (1988). Population dynamics theory—the roles of analytical, simulation, and supercomputer models. Ecol. Model. 43, 111–124 10.1016/0304-3800(88)90075-0

[B50] Piper F. I. (2011). Drought induces opposite changes in the concentration of non-structural carbohydrates of two evergreen nothofagus species of differential drought resistance. Ann. For. Sci. 68, 415–424 10.1007/s13595-011-0030-1

[B51] Powell J. A. Bentz B. J. (2009). Connecting phenological predictions with population growth rates for mountain pine beetle, an outbreak insect. Landscape Ecol. 24, 657–672 10.1007/s10980-009-9340-1

[B52] Powell T. L. Galbraith D. R. Christoffersen B. O. Harper A. Imbuzeiro H. M. A. Rowland L. (2013). Confronting model predictions of carbon fluxes with measurements of Amazon forests subjected to experimental drought. New Phytol. 200, 350–365 10.1111/nph.12390 23844931

[B53] Quirk J. McDowell N. G. Leake J. R. Hudson P. J. Beerling D. J. (2013). Increased susceptibility to drought-induced mortality in *Sequoia sempervirens* (*Cupressaceae*) trees under Cenozoic atmospheric carbon dioxide starvation. Am. J. Bot. 100, 582–591 10.3732/ajb.1200435 23425559

[B54] Rehfeldt G. E. Crookston N. L. Saenz-Romero C. Campbell E. M. (2012). North American vegetation model for land-use planning in a changing climate: a solution to large classification problems. Ecol. Appl. 22, 119–141 10.1890/11-0495.1 22471079

[B55] Rotenberg E. Yakir D. (2010). Contribution of Semi-Arid forests to the climate system. Science 327, 451–454 10.1126/science.1179998 20093470

[B56] Sala A. Piper F. Hoch G. (2010). Physiological mechanisms of drought-induced tree mortality are far from being resolved. New Phytol. 186, 274–281 10.1111/j.1469-8137.2009.03167.x 20409184

[B57] Sitch S. Smith B. Prentice I. C. Arneth A. Bondeau A. Cramer W. (2003). Evaluation of ecosystem dynamics, plant geography and terrestrial carbon cycling in the LPJ dynamic global vegetation model. Glob. Change Biol. 9, 161–185 10.1046/j.1365-2486.2003.00569.x

[B58] Sitch S. Huntingford C. Gedney N. Levy P. E. Lomas M. Piao S. L. (2008). Evaluation of the terrestrial carbon cycle, future plant geography and climate-carbon cycle feedbacks using five Dynamic Global Vegetation Models (DGVMs). Glob. Change Biol. 14, 2015–2039 10.1111/j.1365-2486.2008.01626.x

[B59] Van Nes E. H. Scheffer M. (2005). A strategy to improve the contribution of complex simulation models to ecological theory. Ecol. Modell. 185, 153–164 10.1016/j.ecolmodel.2004.12.001

[B60] Woodward F. I. Cramer W. (1996). Plant functional types and climatic changes: Introduction. J. Veg. Sci. 7, 306–308 10.1111/j.1654-1103.1996.tb00489.x

[B61] Williams A. P. Allen C. D. Macalady A. K. Griffin D. Woodhouse C. A. Meko D. M. (2013). Temperature as a potent driver of regional forest drought stress and tree mortality. Nat. Clim. Change 3, 292–297 10.1038/nclimate1693

[B63] Williams J. W. Jackson S. T. (2007). Novel climates, no-analog communities, and ecological surprises. Front. Ecol. Environ. 5, 475–482 10.1890/070037

[B64] Zeppel M. J. B. Adams H. D. Anderegg W. R. L. (2011). Mechanistic causes of tree drought mortality: recent results, unresolved questions and future research needs. New Phytol. 192, 800–803 10.1111/j.1469-8137.2011.03960.x 22074338

[B65] Zhang L. Dawes W. R. Walker G. R. (2001). Response of mean annual evapotranspiration to vegetation changes at catchment scale. Water Resour. Res. 37, 701–708 10.1029/2000WR900325

